# Rope-Riding Mobile Anchor for Robots Operating on Convex Facades

**DOI:** 10.3390/s25154674

**Published:** 2025-07-29

**Authors:** Chaewon Kim, KangYup Lee, Jeongmo Yang, TaeWon Seo

**Affiliations:** Mechanical Engineering, Hanyang University, 222, Wangsimni-ro, Seoul 04763, Republic of Korea; chae12@hanyang.ac.kr (C.K.); skymusic7@hanyang.ac.kr (K.L.); didwjdah5959@hanyang.ac.kr (J.Y.)

**Keywords:** rope riding, mobile robot, manipulability, linkage mechanism

## Abstract

The increasing presence of high-rise buildings with curved and convex facades poses significant challenges for facade-cleaning robots, particularly in terms of mobility and anchoring. To address this, we propose a rope-riding mobile anchor (RMA) system capable of repositioning the anchor point of a cleaning robot on convex building surfaces. The RMA travels horizontally along a roof-mounted nylon rope using caterpillar tracks with U-shaped grooves, and employs a four-bar linkage mechanism to fix its position securely by increasing rope contact friction. This structural principle was selected for its simplicity, stability under heavy loads, and efficient actuation. Experimental results show that the RMA can support a payload of 50.5 kg without slippage under tensions up to 495.24 N, and contributes to reducing the power consumption of the cleaning robot during operation. These findings demonstrate the RMA’s effectiveness in extending the robot’s working range and enhancing safety and stability in facade-cleaning tasks on complex curved surfaces.

## 1. Introduction

With the growth of industries, cities have also been prospering. As the role of cities grows, the number of buildings to accommodate humans is also increasing. Additionally, advances in construction technology have enabled the construction of taller buildings. This has made high-rise buildings more common in urban areas. High-rise buildings are efficient because these can increase the utilizable space for the same site area as low-rise buildings. Also in the recent past, many high-rise buildings were constructed with simple square-like shapes primarily for economic efficiency. However, modern construction increasingly embraces aesthetics, resulting in more architecturally complex forms such as convex facades.

Owing to this design trend, the number of buildings with irregular shapes is likely to increase consistently. However, the large height of high-rise buildings hinders facade cleaning. The difficulty level increases if the facade is convex rather than flat. The most common approach to cleaning facades is to hire high-rise facade-cleaning workers. Cleaning workers hang from ropes or ride in gondolas to clean the facades. Therefore, the working environment is hazardous, and the safety of cleaning workers is low.

To reduce cleaning costs while reducing workers’ risk, various high-rise facade-cleaning robots and wall climbing mechanisms have been studied [1]. Robots that climb walls using suction cups that generate vacuum include Multi-track [2], NINJA-II [3], Wall Walker [4], and Sky Cleaner series [5]. A suction-type robot can conveniently travel to the desired location on the facade. However, it can be used only on smooth surfaces such as glass and is difficult to be used on rough or convex facades. Robots that climb walls using magnetism include mobile robots with magnetic wheels [6] and TRIPILLAR, which climbs walls using a magnetic caterpillar track [7]. The advantage of magnetic-type wall climbing robots is that these can achieve high speeds on vertical walls. However, there is a limitation that it can be used only on buildings whose facades are composed only of magnetic materials. Robots that climb using wires or ropes have also been developed. ROPERIDE [8] travels vertically along a rope. TITO [9] and Highrise [10,11] also perform vertical movement in a similar manner. However, this method of moving vertically using a rope necessitates a horizontal movement of the rope after each vertical reciprocation to clean the entire facade. Moreover, additional complex external infrastructure installation is required.

As building shapes become increasingly diverse, facade-cleaning robots that can be applied to existing flat facades and to convex facades should be developed. This is because it is difficult to apply the suction-type robot to rough or convex facades and the magnetic type cannot be used unless the facade is magnetic. The rope-type robot was assessed to be appropriate for developing a facade-cleaning robot for use on buildings of all shapes. However, because it can move only vertically, a previous study developed Edelstro-M2. It is a facade-cleaning robot capable of moving in two degrees of freedom using a dual rope [12]. This robot implemented a double-rope climbing mechanism and parallel kinematics by connecting two ropes to two anchors fixed to the top of the building. Consequently, external infrastructure is unnecessary, and it can be applied on any facade by using a pair of ropes. Furthermore, if the anchor can be made to move, when the cleaning robot completes cleaning a certain area, the anchor can be shifted to another area so that the cleaning robot can clean a new area. Additionally, the area where manipulability is low, owing to the tension constraint of the rope, can be reduced. Manipulability implies the capability to reach a certain position or set of positions [13].

**Figure 1:**
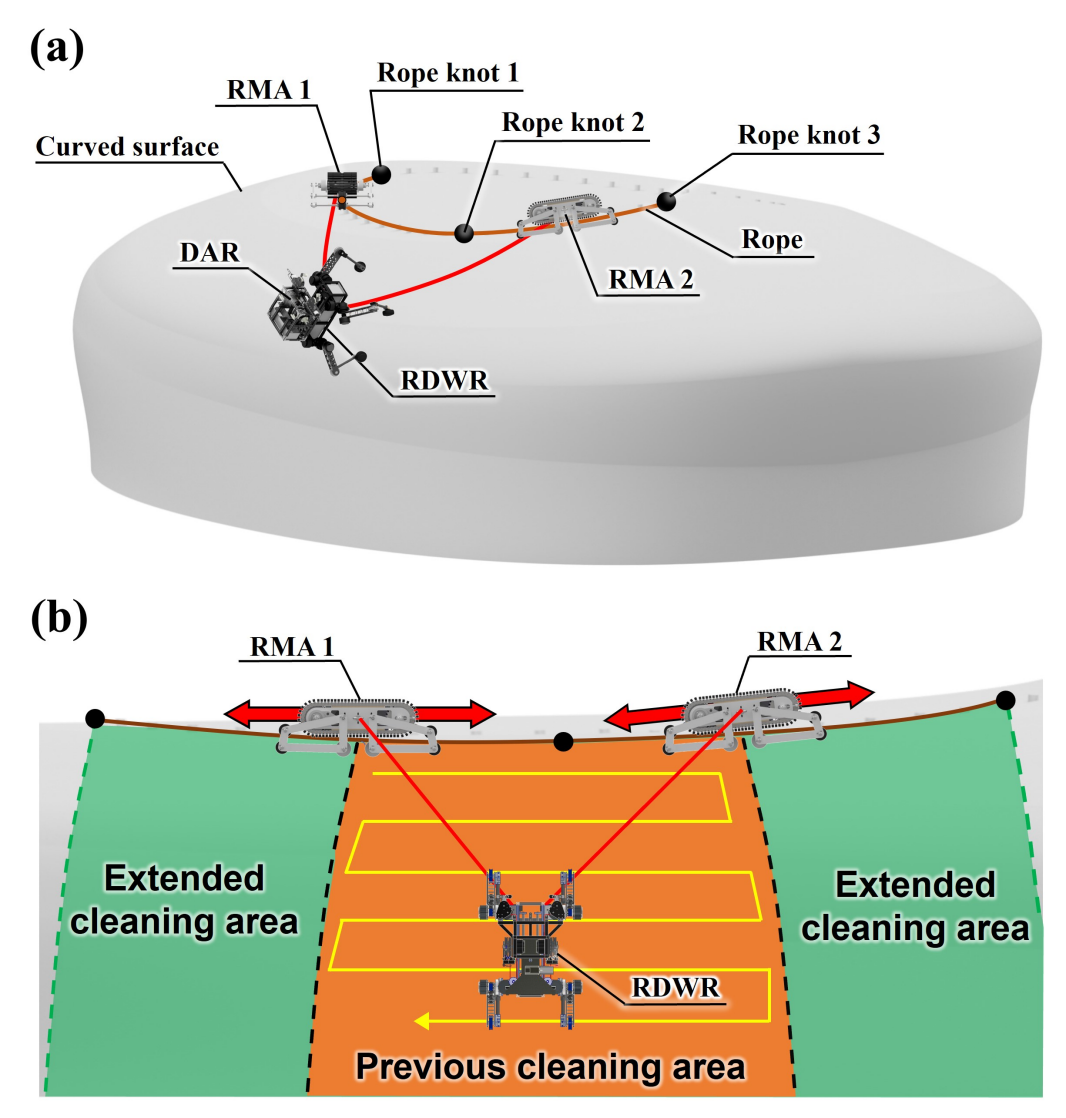
(**a**) Schematics of convex facade cleaning system using RMA. (**b**) Variation in cleaning area when the anchor is fixed and when the RMA is used. The orange area is the cleaning area when the anchor is fixed, and the green area is the extended cleaning area when RMA is used. The RDWR performs cleaning by moving in the direction of the yellow arrow within the cleaning area.

Figure 1a depicts the convex facade cleaning system considered in this study. This system consists of two RMAs: a rope-driven wheel-leg robot (RDWR) and dual ascension robot (DAR) [14]. The RDWR is a four-legged robot with wheels. It overcomes obstacles on a convex facade and cleans it using the cleaning module. The DAR is mounted on the RDWR and is a robot that adjusts the length of the rope [15]. To make the RDWR move in 2-DOF on the facade, the length of the two ropes connecting the RDWR and anchor should be adjusted individually. By the DAR, the RDWR cleans the facade by moving within the cleanable area. RMA works as an anchor in this system to determine the areas that can be cleaned. As depicted in Figure 1b, the RMA rides horizontally on a rope installed on top of a building and moves to another area when the RDWR has completed cleaning a certain area. This significantly improves the cleanable area of the RDWR. Additionally, because the anchor is not fixed to a place and can move left and right on the rope, the manipulability in the same cleaning area is higher than that when the anchor position is fixed. This study focuses on the development of the RMA depicted in Figure 1. So the main contributions of this study are as follows: First, the cleaning area of the cleaning robot can be expanded and the manipulability is increased. Second, since it can be used by simply installing a rope on the building, the cleaning robot system can be easily constructed. Lastly, U-shaped grooved caterpillar tracks enable stable driving with heavy loads on ropes with uneven surfaces.

The structure of the paper is as follows: Section 2 analyzes the variations in DAR manipulability according to the use of the RMA. Section 3 describes the configuration of the RMA. In Section 4, experiments to evaluate the performance of the RMA are described. Finally, Section 5 presents the conclusion of this study.

## 2. Manipulability Analysis of the Cleaning Robot on Convex Facade

**Figure 2:**
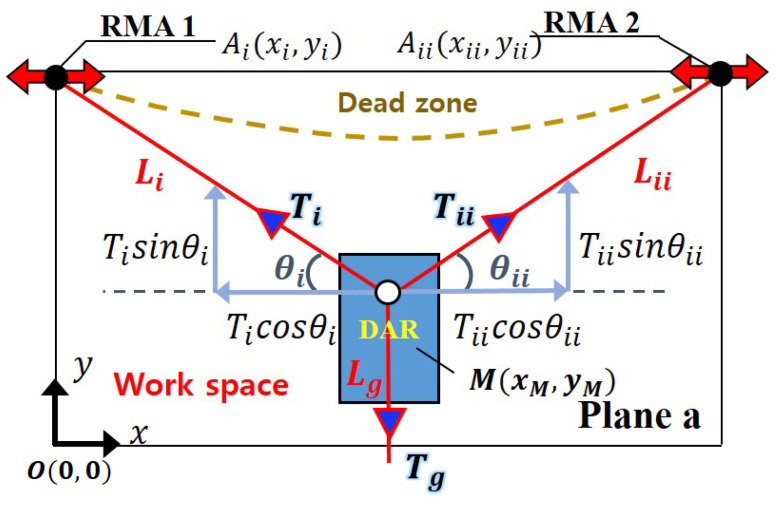
Static analysis of the convex DAR according to the location of the anchor. $L_{i}$, $L_{ii}$, and $L_{g}$ denote the lengths of the ropes connected to RMA 1 and RMA 2 and the virtual gravity length, respectively. $T_{i}$ and $T_{ii}$ are the tensions acting on $L_{i}$ and $L_{ii}$, and $T_{g}$ represents the tension equivalent to the orthogonal projection of the gravitational force $M_{g}$ is orthogonally projected onto the plane *a*. $\theta_{i}$ and $\theta_{ii}$ are the angles formed between the rope and x-axis. $A_{i}$ and $A_{ii}$ represent the x, y coordinates of RMA 1 and RMA 2. *M* represents the x, y coordinates of the DAR. Dead zone is the area that the DAR cannot reach, and work space is the area that it can.

In this section, the manipulability obtained when anchors are fixed on a convex facade and the RMA is used in the same cleaning area is analyzed. In the existing flat facade cleaning system, the workspace of the facade-cleaning robot is limited, owing to the tension constraint of the rope connecting the DAR and anchor [16]. Tension constraint means that as the tension on the rope becomes stronger, the current consumed by the DAR motor, which adjusts the length by winding and unwinding the rope, increases, and when the tension becomes too strong to handle, even with the peak current of the motor, it can no longer move in that direction, which affects the workspace. For the DAR to effectively move horizontally, as depicted in Figure 2, $\theta_{i}$ and $\theta_{ii}$ should be reduced; the rope lengths $l_{i}$ and $l_{ii}$ should increase; and $T_{i}cos\theta_{i}$ and $T_{ii}cos\theta_{ii}$ should increase. Because this reduces the forces $T_{i}cos\theta_{i}$ and $T_{ii}cos\theta_{ii}$ that enable the DAR to move vertically, a dead zone can occur, depending on the motor specifications, below the anchor that the DAR cannot reach. To solve this problem, the development of the movable anchor was considered. If the anchor can move, the DAR can reach all the cleaning areas on a flat facade with only one movable anchor. However, in the case of a convex facade, the DAR spreads radially. This generates areas that are difficult to reach. Therefore, two RMAs should be used so that the DAR can also perform parallel movement. Additionally, in a three-dimensional convex facade, the slope may vary depending on the location. Because of this, the tension of the rope connecting the RMA and DAR varies depending on the location. This, in turn, implies that the DAR may have difficulty reaching certain areas. That is, the manipulability of the DAR decreases in certain areas.

The surface area of Gocheok Sky Dome (GSD) is significantly large. Compared with this, the area of the DAR is significantly small. So it can be assumed that the DAR is located on a plane that is in contact with a point of the convex facade. Moreover, the weight of the 6 mm nylon rope connecting RMA and DAR is approximately 3.58 kg per 100 m. Therefore, the weight of the rope is omitted in this analysis. Figure 2 shows plane *a* describing the schematic of the RMA and DAR suspended on a rope tied to the roof pillar of GSD. To analyze the manipulability according to the location of *M* within the cleaning area, GSD was modeled as a point cloud, and the manipulability at each point was analyzed. Let $\left(x_{M},y_{M}\right)$ denote the coordinates of the DAR, and $\left(x_{i},y_{i}\right)$, $\left(x_{ii},y_{ii}\right)$ be the coordinates of the left and right RMAs, respectively. For a manipulability analysis, coordinate system transformation is performed through the Jacobian matrix *J* [13]. *J* is as follows:

$$\begin{array}{c} J= \end{array}$$

(1) $$\left[\begin{array}{cc} \frac{x_{M}-x_{i}}{\sqrt{{\left(x_{M}-x_{i}\right)}^{2}+{\left(y_{M}-y_{i}\right)}^{2}}} & \frac{y_{M}-y_{i}}{\sqrt{{\left(x_{M}-x_{i}\right)}^{2}+{\left(y_{M}-y_{i}\right)}^{2}}}\\ \frac{x_{M}-x_{ii}}{\sqrt{{\left(x_{M}-x_{ii}\right)}^{2}+{\left(y_{M}-y_{ii}\right)}^{2}}} & \frac{y_{M}-y_{ii}}{\sqrt{{\left(x_{M}-x_{ii}\right)}^{2}+{\left(y_{M}-y_{ii}\right)}^{2}}}\\ 0 & 1 \end{array}\right].$$

The relationship between the rope length matrix *L* and DAR position matrix *M* expressed as *J* is

(2) $$L=\left[\begin{array}{c} L_{i}\\ L_{ii}\\ L_{g} \end{array}\right]\in R^{3\times 1},M=\left[\begin{array}{c} x_{M}\\ y_{M} \end{array}\right]\in R^{2\times 1},\dot{L}=J\dot{M}.$$

**Figure 3:**
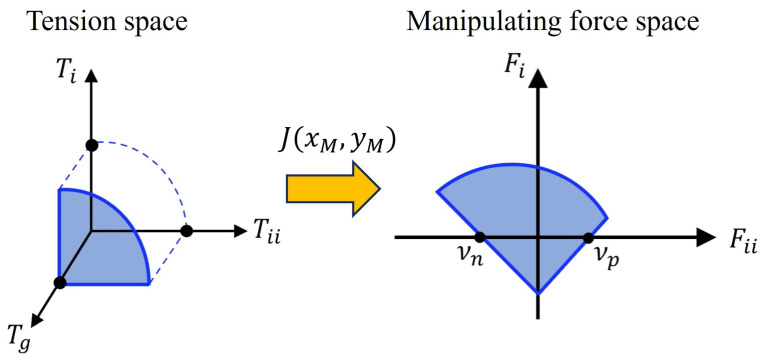
Mapping from tension space to manipulating force space through the Jacobian matrix.

If the force vector by the DAR is *F*, it has the following relationship with the rope tension vector *T*:

(3) $$T=\left[\begin{array}{c} T_{i}\\ T_{ii}\\ T_{g} \end{array}\right]\in R^{3\times 1},F=\left[\begin{array}{c} F_{i}\\ F_{ii} \end{array}\right]\in R^{2\times 1},F=J^{T}T.$$

where $J\in R^{3\times 2}$ maps the velocity of the end-effector position $\dot{M}\in R^{2\times 1}$ to the change rate of rope lengths $\dot{L}\in R^{3\times 1}$. The transpose $J^{T}$ maps the rope tension vector $T\in R^{3\times 1}$ to the resultant manipulating force $F\in R^{2\times 1}$. The tension can be mapped into the manipulating force space by transformation, as depicted in Figure 3.

Facade cleaning can be divided into vertical and horizontal directions. It is relatively convenient to travel in the vertical direction owing to gravity. Therefore, it was decided to analyze the manipulability based on the horizontal direction. Additionally, to prevent obtaining high values in the case where manipulability is zero on either the right or left when the DAR moves, the manipulability ϵ is defined as the product of the positive intercept $\nu_{p}$ and negative intercept $\nu_{n}$ of the $F_{ii}$ axis in Figure 3 [14,17].

(4) $$\epsilon =\nu_{n}\nu_{p}.$$

**Figure 4:**
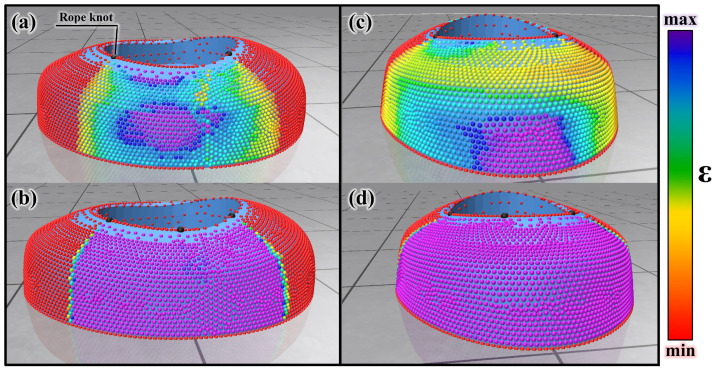
(**a**) Manipulability analyzed from the front area of GSD when the anchor is fixed, which corresponds to F-N in Figure 5. (**b**) Manipulability analyzed from the front area of GSD when using RMA, which corresponds to F-R in Figure 5. (**c**) Manipulability analyzed from the side area of GSD when the anchor is fixed, corresponding to S-N in Figure 5. (**d**) Manipulability analyzed from the side area of GSD when using RMA which corresponds to S-R in Figure 5.

**Figure 5:**
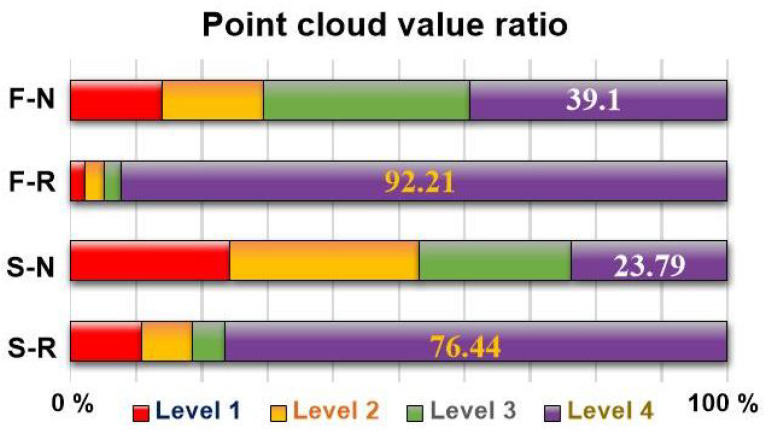
A graph showing the results of Figure 4. The total bar length is set to 100%, and the bar length is assigned according to the proportion of each level.

Figure 4a–d show the manipulability when using fixed anchors and RMA in the front and side areas of GSD. The rope knot in each image refers to the fixed point of the rope used for simulation purposes. In practical implementation, the RMA is capable of moving freely along the rope and locking at arbitrary positions without the need for physical knots or protrusions. The term was used in the simulation context and has been clarified accordingly. The manipulability values are expressed in the order of the colors on the visible spectrum. The value is divided into 20 sections from the minimum to the maximum and displayed in color. The color increases from red to purple, which indicates a higher manipulability. The analysis results show that a higher manipulability is obtained when the RMA is used than when fixed anchors are used. This is depicted in Figure 4. To evaluate the improvement in manipulability, the point cloud value ratio of each front and side area was compared. Figure 5 is a point cloud value ratio graph obtained by the manipulability analysis. ‘F-N’, an abbreviation for ‘Front area Non-RMA’ on the vertical axis denotes the front area when the RMA is not used. ‘F-R’, an abbreviation for ‘Front area using RMA’, denotes the front area when it is used; ‘S-N’, an abbreviation for ‘Side area Non-RMA’, denotes the side area when it is not used; and ‘S-R’, an abbreviation for ‘Side area using RMA’, denotes the side area when it is used. Levels 1, 2, 3, and 4 on the horizontal axis denote the four levels into which the point cloud is divided according to the manipulability value. Color section 1 corresponds to the manipulability value of zero, 2–6 correspond to level 1, 7–11 correspond to level 2, 12–16 correspond to level 3, and 17–20 correspond to level 4. The bar graph represents the proportion of the point cloud corresponding to each level. Level 4 represents the point cloud ratio with the highest manipulability. It increases from 39.1% in F-N to 92.21% in F-R. It also increases from 23.79% when it is S-N, to 76.4% when it is S-R. This implies that using the RMA significantly improves the manipulability for a convex facade.

## 3. Design of the RMA

In Section 2, the need for the RMA was explained through a manipulability analysis. This section describes how the rope-riding mechanism was designed to implement the RMA. Research has been conducted on robots that move on cables or ropes. LineScout [18] uses wheels to move along power transmission lines and perform visual inspection. CCRobot [19] uses grippers to move along pipes. Additionally, robots that use a caterpillar to move on ropes include the caterpillar-based cable climbing robot [20]. The RMA differs from existing rope-riding robots in that it has to move on a rope and do so while hanging from a heavy mass. It should also be convenient to install it on the roof of a building. Therefore, the RMA selected the caterpillar method as the rope riding mechanism. The caterpillar has a wider contact area with the rope than the wheel type or gripper type. So it is more convenient to secure friction for the robot to move and is suitable for running stably on the bumpy surface of the rope. In addition, installation is convenient because a rope can be installed on the roof of a building, and the robot can be used by placing the caterpillar track on the rope. Because a moving heavy object (the RDWR and DAR) is suspended from the RMA, a fixed mechanism was designed to ensure that the RMA is attached effectively to the rope so as to be uninfluenced by disturbances. The RMA can be divided into a driving part for rope-riding and a fixed mechanism part for fixing on the rope. The following describes the design concept and structure of each part.

### 3.1. RMA’s Concept and Configuration

**Figure 6:**
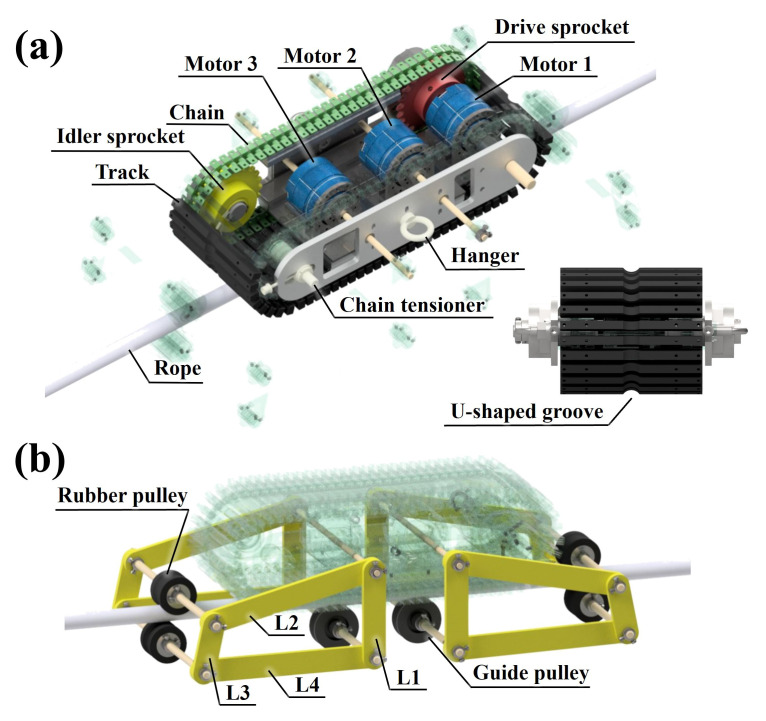
The composition of RMA. (**a**) Its driving part. (**b**) Its fixed mechanism part.

The driving part is depicted in Figure 6a. The overall size of the RMA is 245 mm × 650 mm × 400 mm. For the reasons mentioned above, the RMA selected the caterpillar track as the rope-riding method. The track is made of nitrile-butadiene rubber material to generate sufficient friction. In addition, a U-shaped groove is dug in the center of the track for the RMA to run effectively along the rope. The inside of the RMA cosists of a chain, sprocket, and three motors. The chain maintains an effective tension for caterpillar operation through the chain tensioner. The track is coupled to a chain, and the power supplied from motor 1 is transmitted by a chain sprocket and idler. Motor 1 is for operating the caterpillar track. The remaining two motors are for operating the fixed mechanism. A hanger is placed on the outside of the RMA to suspend the rope connected to the DAR.

### 3.2. Fixed Mechanism’s Concept and Configuration

The fixed mechanism is depicted in Figure 6b. It consists of four links: $L_{1}$, $L_{2}$, $L_{3}$, and $L_{4}$. $L_{1}$ is connected to the guide pulley that fixes the rope and caterpillar track. It guides the driving path. The power provided by the motor is input to $L_{2}$. $L_{3}$ rotates with the movement of $L_{2}$ and is connected to a rubber pulley to generate tension by winding the rope in an S shape. $L_{4}$ connects $L_{3}$ and $L_{1}$. That is, $L_{1}$ is a fixed bar, $L_{2}$ is a crank, $L_{3}$ is a coupler, and $L_{4}$ is a follower.

**Figure 7:**
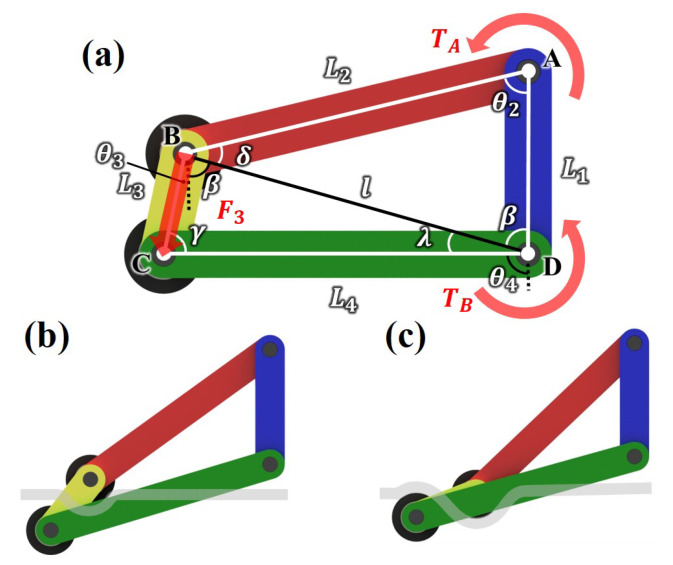
The movement of RMA’s fixed mechanism. (**a**) The variables for analyzing the fixed mechanism. (**b**,**c**) The sequential depiction of the variation in position of the fixed mechanism.

**Figure 8:**
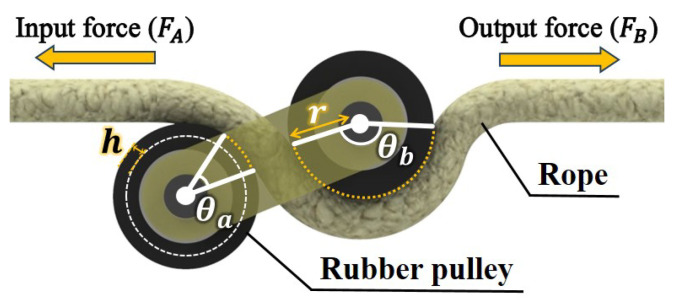
Change in rubber pulley according to the operation of the fixed mechanism, and variation in force according to the capstan equation.

The specific structure and movement of the fixed mechanism are depicted in Figure 7. Figure 7a displays the first position of the fixed mechanism. The rope goes between the two rubber pulleys at $L_{3}$. Figure 7b,c show the position variations of the fixed mechanism when $L_{2}$ moves by the power supplied from the motor. When $L_{2}$ rotates CCW, $L_{3}$ also does so, as depicted in Figure 7b. The rope is wound in an S shape around the rubber pulleys attached to both ends of $L_{3}$ [21]. This is illustrated in detail in Figure 8. If the area where the rope is in contact with the rubber pulley is *A*, the angle by which the rope is wound around the rubber pulley is $\theta_{total}$($\theta_{total}=\theta_{a}+\theta_{b}$, the angle unit is radian), the radius of the rubber pulley is *r*, and the depth of the U-shaped groove of the rubber pulley is *h*, then

(5) $$A=rh\theta_{total},\;\;A_{total}=4A.$$

This generates friction between the rubber pulley and rope. As $\theta_{total}$ increases, the contact area *A* and friction increase. Additionally, the force applied to the rope varies according to Euler’s capstan equation [22]. Additionally, if the input force is $F_{A}$, the output force is $F_{B}$, and the friction coefficient is μ, depending on the variation in wound angle $\theta_{total}$,

(6) $$F_{B}=F_{A}e^{\mu (\theta_{total})}.$$

This enables the fixed mechanism to withstand large forces without slipping, thereby enabling the RMA to be fixed at a specific point on the rope. Figure 7c shows the scenario when $L_{2}$ rotates to its maximum. Because the fixed mechanism is a four-bar linkage structure, attention should be paid to singularity. Therefore, each linkage was calculated so that the fixed mechanism moved only within the range of angles that could prevent singularity. A more detailed analysis is presented in the next subsection.

### 3.3. Fixed Mechanism’s Linkage Analysis

Singularity occurs when $L_{2}$ and $L_{3}$ or $L_{3}$ and $L_{4}$ are aligned while the fixed mechanism is operating. This results in unstable movement of the mechanism, so the input link $L_{2}$ must rotate within the range where singularity does not occur. By analyzing the length combination of each link of the fixed mechanism, an operation range that can prevent singularity should be identified. Moreover, through force analysis, the performance of the fixed mechanism can be improved by predicting which link length combination would produce the highest output torque for an equal input torque. Based on the fixed mechanism depicted in Figure 7 and Figure 8, position static analysis and output torque analysis were performed.

#### 3.3.1. Position Static Analysis

Determine the operating range of the fixed mechanism to prevent singularity. First, draw a line *l* connecting points B and D as shown in Figure 7a. In the triangle ABD, obtained by the law of cosines and law of sines,

(7) $$l=\sqrt{{L_{2}}^{2}+{L_{1}}^{2}-2L_{2}L_{1}\cos\theta_{2}},$$

(8) $$\beta =\arcsin\frac{L_{2}\sin\theta_{2}}{l}.$$

Determine the angle $\alpha (\alpha =\beta +\theta_{3})$ from the triangle BDC,

(9) $${L_{4}}^{2}=l^{2}+{L_{3}}^{2}-2lL_{3}\cos\alpha ,$$

(10) $$\alpha =\arccos\frac{l^{2}+{L_{3}}^{2}-{L_{4}}^{2}}{2lL_{3}},$$

and find λ using the law of cosines and law of sines,

(11) $$\lambda =\arcsin\frac{L_{3}\sin\alpha }{L_{4}}.$$

Find the coordinates of joint *C* based on (7)–(11):

(12) $$C_{x}=-L_{2}\sin\theta_{2}-L_{3}\sin\theta_{3}=-L_{4}\sin\theta_{4},$$

(13) $$C_{y}=-L_{2}\cos\theta_{2}-L_{3}\cos\theta_{3}=-L_{1}-L_{4}\cos\theta_{4}.$$

$C_{x}$ in (12) is the x-coordinate of joint *C*, and $C_{y}$ in (13) is the y-coordinate of joint *C*. Differentiate (12) and (13) one time, and combine these equations. The angular velocity $\omega_{3}$ is

(14) $$\omega_{3}=\frac{-L_{2}\omega_{2}\sin\theta_{2}-\theta_{4}}{L_{3}\sin\theta_{3}-\theta_{4}}.$$

$\omega_{4}$ can be calculated similarly.

To determine the feasible $\theta_{2}$ that do not cause singularity, set the minimum value of $\theta_{2}$ as $\theta_{2}^{min}$, by the second cosine law

(15) $$\theta_{2}^{min}=\arccos\frac{{L_{1}}^{2}+{\left(L_{2}+L_{3}\right)}^{2}-{L_{4}}^{2}}{2L_{1}\left(L_{2}+L_{3}\right)},$$

$\theta_{4}^{min}$, $\theta_{2}^{max}$, and $\theta_{4}{,}^{max}$ can also be calculated similarly. For the fixed mechanism to prevent singularity, $\theta_{2}$ and $\theta_{4}$ should have values only within these ranges.

#### 3.3.2. Output Torque Analysis

Because the input torque provided by the motor had been determined, an analysis was conducted to ensure that the follower link could generate a larger output torque for an equal input torque. γ in Figure 7a can be obtained as follows:

(16) $$\gamma =\arccos\frac{l^{2}-{L_{3}}^{2}-{L_{4}}^{2}}{-2L_{3}L_{4}}.$$

Because the motor torque is known, assuming that the input torque input to $L_{2}$ is $T_{A}$ and the force generated by $L_{3}$ is $F_{3}$,

(17) $$T_{A}=F_{3}L_{2}\sin\left(180-\alpha -\delta \right),$$

(18) $$F_{3}=\frac{T_{A}}{L_{2}\sin\left(180-\alpha -\delta \right)}.$$

Through (16)–(18), the output torque $T_{B}$ can be expressed as follows:

(19) $$T_{B}=F_{3}L_{4}\sin\left(180-\gamma \right)=\frac{T_{A}}{L_{2}\sin\left(180-\alpha -\delta \right)}L_{4}\sin\left(180-\gamma \right).$$

## 4. Experimental Results and Discussion

This section describes experiments conducted to verify that the RMA can achieve the following three goals. First, it should be capable of hanging heavy weights and move on the rope. The sum of the RDWR and DAR weights, which constitute the cleaning system that the RMA should withstand, is 80 kg. Second, it should operate the fixed mechanism on the rope so that it is fixed in the desired position and does not slip while the RDWR and DAR perform cleaning. Lastly, the manipulability of DAR within the cleaning area should be improved. A detailed video showing the robot’s configuration and the full experimental procedure is provided in Supplementary Meterials Video S1.

### 4.1. Capable Payload Experiment

**Figure 9:**
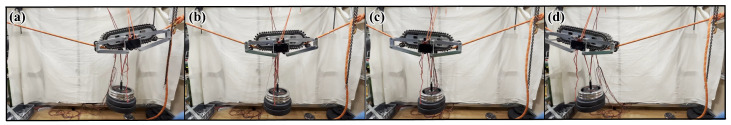
Experiment to determine whether RMA can carry sufficient weight. (**a**–**d**) Sequential RMA movement.

Figure 9 depicts an experiment to evaluate whether the RMA can suspend a sufficient weight and move effectively on the rope. After cleaning a certain area of the facade, the RMA should move on the rope so that other areas can be cleaned. The test bench consists of a 2 m-long rope. The RMA was tested to evaluate whether it can move on the rope with a sufficient payload. Each RMA should be capable of withstanding a payload of at least 40 kg. In the experiment, the RMA could suspend a load of 50.5 kg and move smoothly on the rope. In addition, if the RMA is on a rope alone, the center of mass is located above the rope and may turn over. But if the payload is suspended, the overall center of mass moves below the rope due to its weight, preventing it from overturning. Furthermore, it was verified that the motor torque used in the RMA and the friction force generated between the caterpillar track and rope were sufficient. Although the slope became higher at both ends because of the rope sagging, the RMA displayed stable driving.

### 4.2. Fixed Mechanism Performance Experiment

**Figure 10:**
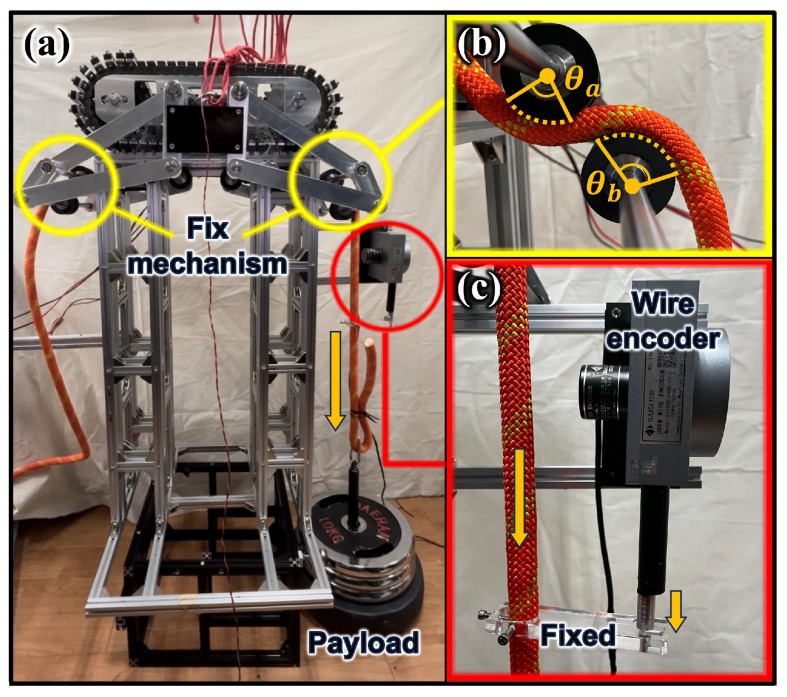
A test bench to evaluate whether a slip occurs in the rubber pulley with payload for verifying the performance of the fixed mechanism. (**a**) The overall configuration of the test bench. (**b**) The operation of the fixed mechanism. (**c**) The wire encoder connected to the rope.

Figure 10a displays a test bench designed to evaluate the performance of the fixed mechanism. The RMA was placed on top of the test bench and operated the fixed mechanism on both sides to wind the rope and generate tension. Figure 10b is an enlarged view of the rubber pulley part of the RMA. As explained in Equation (6), the tension in the rope varied depending on the winding angle. A weight was hung from one side of the ropes. It was tested whether the fixed mechanism could hold the rope so that it did not slip. As depicted in Figure 10c, the rope with the weight was connected to a wire encoder. Therefore, when a slip occurred in the rubber pulley, the rope moved down and the wire encoder detected this. The load of 392.4 N $\left(40\;\mathrm{kg}\times 9.81\;{m/s}^{2}=392.4\;N\right)$ corresponds to the gravitational force generated by a 40 kg payload, which is the expected weight each RMA should support in practical use. In the experiment, a slip did not occur with a load of 495.24 N. This verified that the fixed mechanism has a sufficient rope fixation capability.

### 4.3. Manipulability Experiment

**Figure 11:**
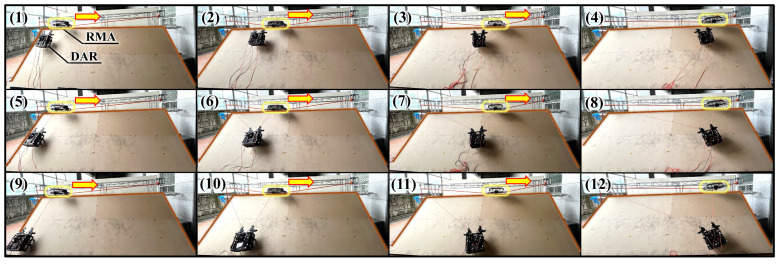
An experiment with DAR moving on an inclined plane using RMA. The yellow arrow indicates the direction in which the RMA moves on the rope. Numbers (1)–(12) written in the photograph represent the experiment conducted by locating the DAR at each test point.

As presented in Section 2, the manipulability was calculated using a cloud point model of GSD, and the results were explained. Based on this, it was most effective to test the RMA on the actual building. However, it was considered hazardous to conduct an actual field test because the convex facade cleaning system had not been fully established and could have caused safety issues. Meanwhile, the area occupied by the DAR is significantly smaller than the actual area of GSD. Therefore, the analysis was feasible while assuming it to be flat, as shown in Figure 2. So a test bench was constructed with an inclined plane corresponding to the contact surface generated when the DAR was located at a certain point on GSD. The test bench has a height and width of 2 m and 6 m, respectively, and includes an inclined plane. There are 12 test points on the inclined plane ((1)–(12)). At each test point, the DAR’s motor current was compared between the case without using RMA and that using RMA. The less current the motor uses, the better manipulability it has. Figure 11 depicts the experiment conducted using the RMA. A rope is installed between the two ends of the top of the test bench, and the RMA is installed on it. One rope of the DAR is connected to the top of a pillar of the test bench, and the other rope is connected to the RMA. The RMA moves left and right on the rope and searches for the fixed location that requires the least amount of DAR motor current at each test point. In the case of the experiments without the RMA, a rope connected to the DAR was fixed to the top of the pillars at both ends of the test bench, and the motor current of the DAR was measured at each test point.

**Figure 12:**
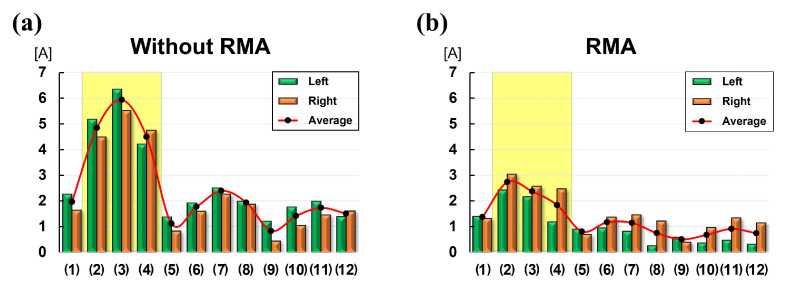
Graph showing experimental results obtained with and without RMA use. The x-axis corresponds to the test points in Figure 11. The y-axis represents the current consumption of the motors on both sides of the DAR at each test point. (**a**) Graph showing the results obtained when RMA is not used. (**b**) Graph showing the results obtained when RMA is used.

The experimental results are shown in Figure 12. Figure 12a shows the value of the current utilized by the left and right motors of the DAR at each test point when the RMA was not used. Figure 12b shows the value of the current utilized by the left and right motors of the DAR at each test point when the RMA was used. This is the result of Figure 11. The green bar graph represents the current consumption of the left motor of the DAR, the orange bar graph represents the current consumption of the right motor of the DAR, and the red curved graph represents the average of the two current consumption values. Comparing the results, overall, all the test points consumed less current with the RMA than without it. In particular, the difference in current consumption depending on whether the RMA was used or not was the largest in the test point depicted in Figure 12(2)–(4), and is in the section indicated by the yellow area. This is because $\theta_{i}$ and $\theta_{ii}$ were reduced, as explained in Figure 2 in Section 2. If the RDWR is also applied, the current difference would be larger because the load on the RMA would be larger. This implies that dead zones that may occur depending on motor specification can be reduced using an RMA.

## 5. Conclusions

In this study, a robot RMA that modifies the cleaning area of a convex facade cleaning system was developed. The RMA consists of a driving part and a fixed mechanism part. This enables the RMA to be secured on the rope and function as an anchor when the cleaning systems perform convex facade cleaning. The fixed mechanism has a four-bar linkage structure and uses the capstan equation to strengthen the tension on the rope. This enables the RMA to remain stationary without slipping. The RMA improves the manipulability of the DAR. The RMA-performance test revealed the feasibility of riding on a rope with a payload of 50.5 kg per RMA. Moreover, in the case of the fixed mechanism, a slip did not occur in the rubber pulley even with a pulling force of 495.24 N. Therefore, it is concluded that it has a sufficient capability to shift the operating area of the cleaning system on the convex facade. In addition, it was verified that the RMA improves the manipulability because the current consumed by the DAR motor at an equal test point is less than that when RMA is not used. In particular, the upper part of the cleaning area significantly reduces the current used. Therefore, the dead zone problem can be solved using the RMA.

In this paper, The RMA is designed for implementing the movement system of the convex-facade-cleaning robots. However, there is a limitation that only analysis and laboratory tests in a controlled environment were performed. In future studies, the field tests and stability analysis by the field test that actually installing the RMA on the convex facade.

## 6. Limitations and Future Work

The proposed rope-riding mobile anchor (RMA) system experimentally demonstrated that it can effectively support a cleaning robot on a convex outer wall. However, there are still some limitations that need to be addressed, which will be supplemented in future studies.

First, the elongation of the rope due to tension was not included in the analysis model in this study. In a large-scale environment, the length of the rope increases, and the elongation caused by this can affect the robot’s operability and positioning accuracy. Accordingly, in future studies, we plan to analyze the effect of the elasticity of the rope on the control performance by reflecting it in the dynamic model.

Second, the influence of external environmental factors (especially wind) was not considered in the experiment. In a real environment, vibration or additional load due to wind can impede the stability of the system, and a closed-loop control strategy is required to compensate for this. These factors will be examined in future outdoor full-scale tests.

Third, the current system is designed for outer walls with vertical or positive slopes. However, in some buildings, there may be a local negative slope (underhang), and in this case, the robot may be detached from the outer wall due to gravity. To solve this problem, future research will additionally design an adsorption-type fixed attachment mechanism or a hybrid fixation technology.

Fourth, this study focused on verifying the mechanical structure and load-bearing capacity of the RMA system, but in actual applications, cleaning speed measurement, path-tracking control, and system power consumption analysis are essential. These system-level performance factors will be addressed in an environment integrated with the cleaning module in the future.

Finally, the experiments in this study were conducted in a scaled-down model environment. In an actual building environment, various factors such as rope sagging, long-distance control signal transmission, and maintenance accessibility should be additionally considered. Therefore, evaluating the practicality and durability of the system through large-scale field experiments based on actual buildings will be an important task for future research.

## Data Availability

The raw data supporting the conclusions of this article will be made available by the authors on request.

## Supplementary Materials

The following supporting information can be downloaded at: https://www.mdpi.com/article/10.3390/s25154674/s1, Video S1: “Rope-Riding Mobile Anchor for Robots Operating on Convex Facades” contains concepts and experiment videos.
